# Regulation of Mitochondrial and Peroxisomal Metabolism in Female Obesity and Type 2 Diabetes

**DOI:** 10.3390/ijms252011237

**Published:** 2024-10-19

**Authors:** Damián A. Antelo-Cea, Laura Martínez-Rojas, Izan Cabrerizo-Ibáñez, Ayda Roudi Rashtabady, María Isabel Hernández-Alvarez

**Affiliations:** 1 Departament de Bioquímica i Biomedicina Molecular, Facultat de Biologia, Universitat de Barcelona, 08028 Barcelona, Spain; damian.antelo.cea@gmail.com (D.A.A.-C.); lala.mrojas@gmail.com (L.M.-R.); izancabrerizo7ub@gmail.com (I.C.-I.); roudi.r@gmail.com (A.R.R.); 2 IBUB Universitat de Barcelona—Institut de Biomedicina de la Universitat de Barcelona, 08028 Barcelona, Spain; 3 Centro de Investigación Biomédica en Red de Diabetes y Enfermedades Metabólicas Asociadas (CIBERDEM), Instituto de Salud Carlos III, 28029 Madrid, Spain

**Keywords:** obesity, type 2 diabetes, lipid metabolism, peroxisomes, estrogens, PPARs, insulin resistance, metabolic disorders

## Abstract

Obesity and type 2 diabetes (T2D) are widespread metabolic disorders that significantly impact global health today, affecting approximately 17% of adults worldwide with obesity and 9.3% with T2D. Both conditions are closely linked to disruptions in lipid metabolism, where peroxisomes play a pivotal role. Mitochondria and peroxisomes are vital organelles responsible for lipid and energy regulation, including the β-oxidation and oxidation of very long-chain fatty acids (VLCFAs), cholesterol biosynthesis, and bile acid metabolism. These processes are significantly influenced by estrogens, highlighting the interplay between these organelles’ function and hormonal regulation in the development and progression of metabolic diseases, such as obesity, metabolic dysfunction-associated fatty liver disease (MAFLD), and T2D. Estrogens modulate lipid metabolism through interactions with nuclear receptors, like peroxisome proliferator-activated receptors (PPARs), which are crucial for maintaining metabolic balance. Estrogen deficiency, such as in postmenopausal women, impairs PPAR regulation, leading to lipid accumulation and increased risk of metabolic disorders. The disruption of peroxisomal–mitochondrial function and estrogen regulation exacerbates lipid imbalances, contributing to insulin resistance and ROS accumulation. This review emphasizes the critical role of these organelles and estrogens in lipid metabolism and their implications for metabolic health, suggesting that therapeutic strategies, including hormone replacement therapy, may offer potential benefits in treating and preventing metabolic diseases.

## 1. Introduction

**Figure 1:**
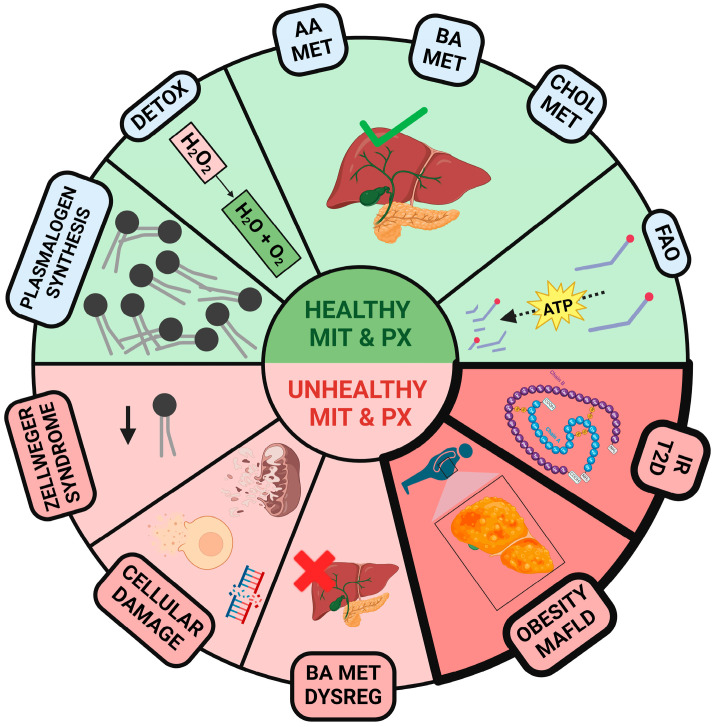
The green section presents a general overview of various metabolic pathways involving healthy mitochondria (MIT) and peroxisomes (PX). These include plasmalogen synthesis, detoxification of hydrogen peroxide (H_2_O_2_) into H_2_O and O_2_, amino acid (AA) metabolism, bile acid (BA) metabolism, cholesterol (CHOL) metabolism, and fatty acid oxidation (FAO). The red section highlights various diseases associated with dysfunctional MIT and PX, such as Zellweger syndrome, which involves multiple genetic PX disorders, cellular damage, dysregulation of BA metabolism, the development of obesity, metabolic dysfunction-associated fatty liver disease (MAFLD), insulin resistance (IR), and type 2 diabetes (T2D). Created with www.Biorender.com.

Through oxidative phosphorylation, mitochondria generate the majority of cellular adenosine triphosphate (ATP) and are involved in fatty acid β-oxidation [1]. Peroxisomes are single-membrane-bound organelles that play crucial roles in cellular metabolism. Lacking DNA or ribosomes, these organelles house catalase and several oxidative enzymes involved in critical catabolic and anabolic processes, such as the α- and β-oxidation of fatty acids, glyoxylate detoxification, and the degradation of purines and polyamines [2]. Through these processes, peroxisomes regulate vital metabolic pathways, including cholesterol synthesis, bile acid production, and lipoprotein metabolism [3,4] (Figure 1). These organelles often work in tandem with mitochondria, another essential organelle responsible for energy production and cellular metabolism. While peroxisomes initiate the β-oxidation of very long-chain fatty acids, mitochondria complete their degradation, highlighting the metabolic interplay between these organelles [5]. Mitochondrial efficiency also plays a critical role in maintaining insulin sensitivity and regulating lipid metabolism, further underscoring the importance of coordinated function between these organelles in preventing metabolic imbalance [6]. Disruptions in peroxisomal–mitochondrial function and crosstalk can significantly impact lipid metabolism, leading to metabolic imbalances associated with obesity and type 2 diabetes (T2D) [6,7] (Figure 1), two diseases that significantly impact global health today, affecting approximately 17% of adults worldwide with obesity and 9.3% with T2D [8,9].

In individuals with obesity, defective fatty acid oxidation may result in the accumulation of lipids, exacerbating insulin resistance and chronic inflammation, both of which are key factors in the pathophysiology of T2D. Additionally, peroxisomal dysfunction has been linked to increased oxidative stress and lipid peroxidation, further aggravating metabolic complications [10]. Proper regulation of these metabolic pathways is also influenced by hormonal factors, particularly estrogen, a crucial hormone in women that modulates lipid metabolism and insulin sensitivity throughout different phases of the menstrual cycle, fertility, and menopause [11].

This review aims to explore the intersection between mitochondria–peroxisome dysfunction and estrogen-mediated hormonal regulation, focusing on exposing potential therapeutic targets for treating metabolic diseases, such as obesity and type 2 diabetes.

## 2. Estrogen Influence in Liver Metabolic Dysfunction, Obesity, and T2D

Metabolic dysfunction-associated fatty liver disease (MAFLD) poses a significant global health challenge and is closely linked to conditions such as obesity, insulin resistance, hypertriglyceridemia, and T2D. The progression of MAFLD facilitates the differentiation from early-stage steatosis to more severe conditions, such as metabolic dysfunction-associated steatohepatitis (MASH). A key pathological feature of MASH is fibrosis, which results from an excessive accumulation of extracellular matrix (ECM) components, including collagens type I, III, and IV, as a hallmark of the disease [12]. Hepatic stellate cells (HSCs) play a crucial role in the progression of liver disorders, as they differentiate into myofibroblast-like cells that express α smooth muscle actin (α-SMA) [13].

Interestingly, men are more prone to developing MAFLD than women, which has sparked research into the potential protective role of estrogen [14]. Although estrogen is predominantly known as a female hormone, it is also present in men at lower levels and plays a crucial role in regulating sexual function, as well as various other physiological processes, including energy balance [15]. In women, the three main endogenous estrogens—estriol (E3), 17β-estradiol (E2), and estrone (E1)—are synthesized from cholesterol, with E2 being the most potent and dominant in premenopausal women [16]. While the gonads are the primary source of E2, it is also produced in the liver, brain, adrenal glands, and adipose tissue. After menopause, ovarian E2 production declines, making E1 the dominant form, which primarily serves as a precursor to E2 [17,18]. The reduced E2 production post-menopause has been investigated in mouse models, revealing significant metabolic alterations, including the onset of dyslipidemia, fatty liver, and insulin resistance in female mice [19]. Additionally, a recent review contrasted the onset of menopause with its impact on insulin resistance and the prevalence of MAFLD [20]. The findings indicate that women experience a higher prevalence of MAFLD and increased insulin resistance, primarily due to hypoestrogenism during this stage. These hormonal changes, alongside alterations in body composition associated with menopause, contribute to the complexity and multidimensional nature of metabolic syndrome development [20].

The liver plays a crucial role not only in maintaining metabolic homeostasis but also as a critical site for estrogen action through its receptors. In the body, estrogen acts via two main types of receptors: membrane receptors, such as G-protein-coupled estrogen receptors, and nuclear receptors, primarily ERα and ERβ [21]. Among these, ERα is the predominant receptor in the liver, essential for regulating metabolic processes. Since insulin resistance is closely linked to hepatic steatosis, understanding estrogen’s influence on liver function is vital for developing effective treatments for metabolic disorders.

Research using low- or no-estrogen models, such as aromatase knockout mice, has provided valuable insights into the relationship between estrogen and liver metabolism. These studies show that the absence of estrogen leads to metabolic dysregulation, marked by elevated insulin levels and increased fat accumulation, primarily due to reduced glucose oxidation. Notably, males experience significantly higher insulin resistance, a condition that can be improved with estrogen therapy, underscoring the potential therapeutic role of estrogens in treating metabolic disorders [22].

Furthermore, studies involving male Wistar rats with liver fibrosis induced by dimethylnitrosamine (DMN) have demonstrated the antifibrogenic effects of estradiol. These studies found that estradiol treatment resulted in lower mRNA levels of type I and III procollagens and decreased α-SMA expression, inhibiting liver fibrosis progression. In contrast, anti-estradiol antibody therapy exacerbated liver fibrosis, underscoring the importance of estrogen in maintaining liver health [13].

Moreover, the liver is considered the primary site of estrogen degradation due to its abundance of cytochrome P450 enzymes [23]. There are two main pathways in which the body metabolizes estradiol and estrone, the two active forms of estrogen, principally through the action of CYP1A2 and CYP1B1 enzymes. CYP1A2 accounts for approximately 80% of estradiol metabolism, while CYP1B1 accounts for the remaining 20%. Additionally, research by Tsuchiya and colleagues has revealed a link between reduced estrogen degradation by CYP1A2 and increased activity by CYP1B1 with DNA damage and genotoxic carcinogenesis, a condition more prevalent in men [23,24]. In this context, estradiol metabolites, particularly 2-methoxyestradiol, have demonstrated potential anticancer properties, though these effects appear to function independently of estrogen receptors ERα and ERβ [23,25].

A recent clinical trial by Konopka et al. (2015) on mitochondrial dysfunction in obese, insulin-resistant women with polycystic ovary syndrome (PCOS) demonstrated how impaired mitochondrial coupling and elevated hydrogen peroxide (H_2_O_2_) emissions exacerbate oxidative stress and metabolic dysregulation. Interestingly, this dysfunction closely mirrors the estrogen-deficient state observed in post-menopausal women and those with metabolic syndrome, where mitochondrial inefficiency contributes to reduced fatty acid oxidation and increased lipid accumulation in the liver [26].

The clinical trial highlights the potential of aerobic exercise to reverse mitochondrial impairments and restore a “lean” mitochondrial phenotype in these women. Aerobic exercise is known to enhance estrogen signaling pathways and metabolites [27], leading to improved mitochondrial phosphorylation efficiency and decreased mitochondrial H_2_O_2_ emissions. This suggests that interventions targeting mitochondrial bioenergetics may help counteract estrogen deficiency-induced metabolic dysfunctions in obesity and T2D.

Peroxisomes, as the cell’s primary redox regulators [28], have been the focus of research into their potential crosstalk with mitochondria through the detoxification of reactive oxygen species (ROS), such as H_2_O_2_, for several years [29]. Emerging evidence suggests that peroxisomes play a crucial role alongside mitochondria in regulating ROS and that changes in the peroxisomal redox state are likely to influence mitochondrial redox activity [30].

Estrogen plays a multifaceted role in liver metabolic dysfunction, obesity, and diabetes, influencing various pathways involved in metabolic regulation and liver health. Understanding these interactions is crucial for developing targeted therapies that leverage estrogen’s protective effects against metabolic diseases.

## 3. PPAR Involvement in Obesity through β-Oxidation of Fatty Acids

### 3.1. Biogenesis and PPARs Function

Peroxisome biogenesis involves the de novo formation of peroxisomes from pre-existing organelles or ER-derived vesicles. This process is orchestrated by a group of specialized proteins known as peroxins, encoded by peroxin (PEX) genes, which are vital for the formation, maintenance, and functionality of peroxisomes [31,32,33].

The regulation of peroxisome biogenesis and proliferation is intricately linked to cellular signaling pathways that respond to environmental cues, such as alterations in lipid levels and oxidative stress or metabolic demands [34].

**Figure 2:**
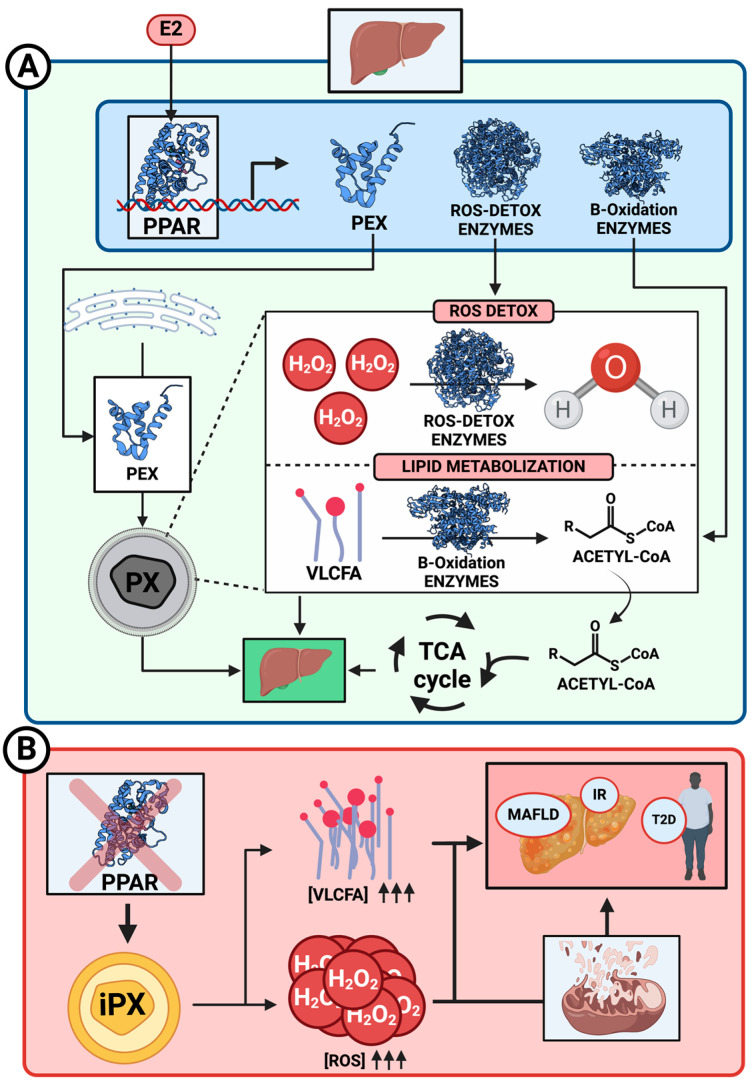
(**A**) Illustration of the various roles of peroxisome proliferator-activated receptors (PPARs) in normal peroxisomes (PX) under healthy liver conditions. PPARs are regulated by 17β-estradiol (E2) and activate the transcription of proteins involved in essential PX functions: peroxins (PEX) support PX biogenesis; reactive oxygen species (ROS) detoxification enzymes, such as catalases, and facilitate the detoxification of H_2_O_2_; and β-oxidation enzymes metabolize very long-chain fatty acids (VLCFAs) into acetyl-CoA, which is then transported to mitochondria and incorporated into the Krebs cycle (TCA) to generate adenosine triphosphate (ATP). (**B**) Overview of the consequences of PPAR dysfunction. This impairment results in defective PX (iPX) function, leading to the accumulation of VLCFA and ROS, which compromise mitochondrial stability and contribute to the development of insulin resistance (IR), type 2 diabetes (T2D), and metabolic dysfunction-associated fatty liver disease (MAFLD). Created with www.Biorender.com.

The peroxisome proliferator-activated receptors (PPARs) directly mediate this proliferation and lipid metabolism. These nuclear receptors PPAR-α, PPAR-β, and PPAR-γ can induce peroxisome and mitochondrial proliferation, the last one principally by the activation of peroxisome proliferator-activated receptor-gamma coactivator-1α (PGC-1α), and can upregulate genes encoding both organelles’ enzymes, enhancing the organelles’ capacity to metabolize lipids and detoxify ROS (Figure 2), illustrating the adaptive response of peroxisomes and mitochondria to metabolic changes. A dysfunction of this metabolism could lead to different diseases principally related to the accumulation of lipids, which directly affects the liver, resulting in the development of the first steps of MAFLD [35,36].

### 3.2. Fatty Acid β-Oxidation and MAFLD Generation

Fatty acid β-oxidation in peroxisomes is a mechanism to promote an alternative source of energy in the form of Acetyl-CoA, which is finally incorporated into the Krebs cycle to produce ATP in mitochondria (Figure 2A).

This special fatty acid β-oxidation is essential in mammals to degrade long-chain fatty acids, which cannot be oxidated in mitochondria. Shortened long-chain fatty acids are further degraded by the mitochondrial β-oxidation pathway to produce energy. This pathway is activated during fasting periods or high energy demand states, such as exercise [37,38,39].

Different human disorders have been associated with the dysfunction of peroxisomes, which commonly leads to elevated levels of very long-chain fatty acids (VLCFAs) in plasma, which are directly associated with β-oxidation.

X-linked adrenoleukodystrophy (X-ALD) leads to impaired VLCFA metabolism, specifically reducing C26:0 β-oxidation to about 30% of normal levels. This impairment results in the accumulation of VLCFA-CoA esters in cells due to mutations in the ABCD1 transporter linked to the X chromosome. This primarily causes the accumulation of fatty acids in the brain, leading to various neurological issues [40].

Others, such as the 2-Methylacyl-CoA racemase deficiency, cause a defect in branched-chain fatty acid ß-oxidation, leading to an accumulation of branched-chain fatty acids [41].

The accumulation of fats, primarily in the liver, can eventually lead to MAFLD, which may progress to a state of steatohepatitis known as metabolic dysfunction-associated steatohepatitis (MASH) (Figure 2B). This type of fatty liver is associated with various complications, including insulin resistance and obesity.

Different articles have comprehensively reviewed these disorders, highlighting the clinical relevance of proper peroxisome biogenesis and the importance of understanding the molecular mechanisms underlying these processes to develop potential therapeutic strategies [42]. Studies have confirmed that dysfunctional peroxisomes and mitochondria can significantly impair lipid metabolism, leading to conditions such as fatty liver, hypercholesterolemia, and atherosclerosis. For example, when peroxisomal β-oxidation, an essential pathway in fatty acid metabolism, is disrupted, it can result in the accumulation of fatty acids and cholesterol intermediates, contributing to metabolic disorders directly affecting the mitochondrial function [7,43,44]. For instance, a defect in enzymes such as Acox1, critical for peroxisomal fatty acid oxidation, has been linked to MAFLD and hepatic inflammation, exacerbating liver damage over time in part due to the higher mitochondrial biogenesis and fatty acid β-oxidation that takes place as a compensatory effect, which may lead to oxidative stress and ROS accumulation (Figure 2B) [43,44,45].

This metabolic dysfunction, paired with impaired bile acid production, can disrupt cholesterol balance, making individuals more susceptible to lipid accumulation and oxidative stress [45,46].

### 3.3. E2 Regulation of PPARs

The family of estrogen hormones, represented principally by the 17β-estradiol (E2), is known to influence lipid metabolism by modulating enzymes, genes, and pathways involved in lipid synthesis, storage, and oxidation, especially in the liver [11]. All these include indirect effects on peroxisomes and mitochondria through PPARs.

Estrogen’s regulation of PPAR-γ and PPAR-α in liver diseases promotes fatty acid oxidation rates, with potential implications for peroxisome numbers and functionality. PPARs are involved in the metabolism of triacylglycerides and the regulation of energetic homeostasis by controlling glucose levels, insulin function, and fatty acid beta-oxidation through different genetic modulations (Figure 2A). The regulation of pathways through PPARs by E2 directly impacts the onset of T2D, obesity, and fatty liver when PPARs are dysfunctional, primarily due to insulin resistance in the early stages of these diseases [47,48,49,50].

Currently, research is already being conducted on utilizing PPARs as therapeutic targets to restore function. Scientists are investigating the use of PPARγ agonists to activate antioxidant and anti-inflammatory effects in neuronal cells as potential mitochondria-targeted therapies, aiming to prevent the onset of conditions such as Parkinson’s and Alzheimer disease [51].

Some studies indicate that PPAR-α can increase insulin function, inhibiting β-oxidation in peroxisomes. In dysfunctional peroxisomes, changes in PPAR-α modulation increase the production of fatty acids while decreasing insulin sensitivity, leading to the development of obesity and T2D. 

Various research papers by Yoon and colleagues in 2009 and 2010 indicated that increased E2 levels in male livers could potentially treat obesity caused by PPAR dysregulation, although it remains uncertain whether it can address obesity in females with intact ovaries [48,49].

E2 acts through different estrogen receptors in the cell. The main one, anchored to the plasma membrane, has two subunits (ER-α and ER-β) that function by forming a signaling system coupled to a G-protein, producing the signaling cascade effect by phosphorylation of the G-protein subunits. On the other hand, nuclear estrogen-related receptors exist, namely, ERRα, ERRβ, and ERRγ. Taking into account that ERRα is the primary player in this receptor family, its central role is to coactivate PGC-1α/ß, leading to the transcription of various genes related to the TCA cycle, fatty acid β-oxidation, oxidative phosphorylation (OXPHOS), and vascular endothelial growth factor (VEGF) [11,52,53].

The interaction between estrogen and PPARs is in continuous study. Estrogens are known to influence the modulation of PPAR, but the pathway is not well known. Some papers talk about a direct modulation of PPAR-γ by the ER-α/β system in the cytosol and the possibility of the existence of crosstalk between both proteins; in this case, the expression of one will be regulated by the action of the other [50].

On the other hand, other studies explain the possibility of a nuclear interaction between ERRα and PPAR-α, also enhancing its function with the action of the PGC-1α/ß nuclear cofactor as the same with other transcriptional regulators of mitochondrial genes, such as the nuclear respiratory factor 1 (NRF1) or the GA-binding protein alpha (GABPα) subunit of GABP/NRF2 (GA-binding protein/nuclear respiratory factor 2) in heart or brown adipose tissue (BAT) [52]. Finally, the impact of hormones was assessed by monitoring the restoration of balance in fatty acid processing in fat cells following estradiol treatment [54,55].

## 4. Cholesterol Synthesis Regulation

Cholesterol synthesis is a vital metabolic pathway in cells, particularly in the liver, involving mitochondria, peroxisomes, and the endoplasmic reticulum (ER). This process is regulated through a feedback mechanism to maintain proper cholesterol levels and prevent excessive accumulation. 3-hydroxy-3-methyl-glutaryl-coenzyme A reductase (HMG-CoA reductase) plays a crucial role in the early stages of cholesterol synthesis by converting HMG-CoA to mevalonate, which is a rate-limiting step in the process. This enzyme is the primary target for feedback regulation [56,57,58,59].

When intracellular cholesterol levels are high, the body downregulates the activity of HMG-CoA reductase to slow down cholesterol production. This regulation occurs principally through the action of sterol regulatory element-binding proteins (SREBPs), transcription factors that control the expression of HMG-CoA reductase. High cholesterol levels trap SREBP in the endoplasmic reticulum, preventing it from entering the nucleus and activating genes for cholesterol synthesis enzymes [60,61].

### 4.1. Implication of Peroxisomes–Mitochondria in Cholesterol Synthesis

A new potential pathway for cholesterol synthesis has recently been proposed, highlighting the role of peroxisomes as a key intermediate organelle in this metabolic process [61]. The authors explain the possibility that peroxisomes have their own enzymes with the capacity to metabolize VLCFA to HMG-CoA and, after that, to mevalonate, a key intermediate in the cholesterol synthesis pathway [56].

**Figure 3:**
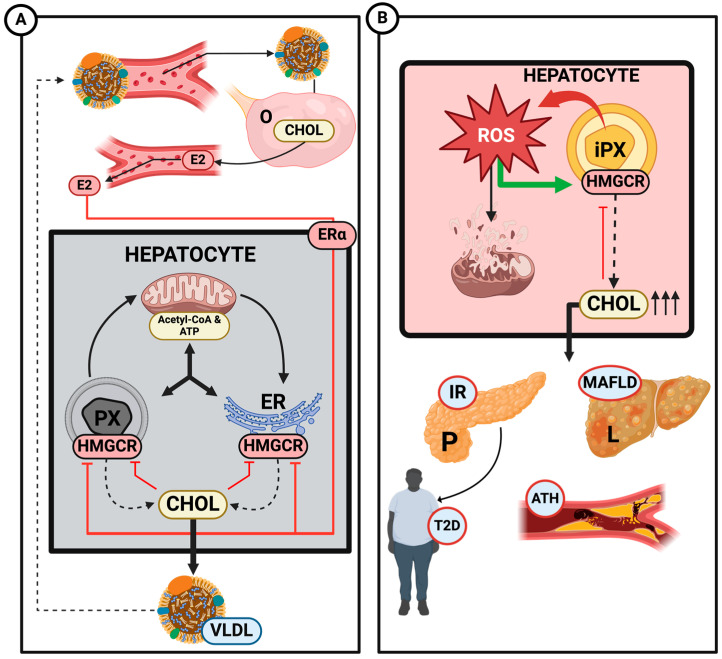
(**A**) Diagram of endogenous cholesterol synthesis and its regulation by 17β-estradiol (E2) in hepatocytes. Peroxisomes (PX), mitochondria, and the endoplasmic reticulum (ER) interact to produce cholesterol (CHOL). HMG-CoA reductase (HMGCR) functions as the rate-limiting enzyme in this process. It is regulated by E2 through its estrogen receptor-α (ERα) and SREBP, depending on the circulating cholesterol levels, forming a negative feedback regulation. The cholesterol produced is utilized in various pathways, including transport to the ovaries (O) for E2 synthesis. (**B**) Different consequences of dysregulated cholesterol pathways due to impaired PX (iPX) in hepatocytes. iPX in hepatocytes increases cholesterol production by upregulating enzymes like HMGCR while simultaneously downregulating detoxifying enzymes like catalases. This imbalance results in the accumulation of cholesterol and fatty acids, contributing to insulin resistance (IR), the development of type 2 diabetes (T2D), metabolic dysfunction-associated fatty liver disease (MAFLD), and atherosclerosis (ATH). Additionally, the loss of the PX detoxification capacity leads to reactive oxygen species (ROS) accumulation, directly affecting the mitochondria, elevating oxidative stress levels, and causing organelle disruption. Created with www.Biorender.com.

The pathway begins with acetyl-CoA, derived from the β-oxidation of fatty acids in the mitochondria, which is then transported to the ER to produce mevalonate. Simultaneously, mevalonate produced in the ER is transported to the peroxisomal matrix for further conversion into isopentenyl pyrophosphate (IPP). This IPP is then transported back to the ER via the ACBD5-VAP transporter, where cholesterol synthesis is completed (Figure 3A) [61,62].

The cholesterol synthesized in the cell serves several vital functions: it can be esterified to form lipoproteins, such as very-low-density lipoprotein (VLDL) and high-density lipoprotein (HDL), which are critical for lipid transport throughout the body. Cholesterol also plays a role in bile acid production, a process that requires its entry into peroxisomes for further enzymatic reactions [3]. Additionally, cholesterol is transported to the ovaries, which act as a precursor for synthesizing steroid hormones, including estrogens. Lastly, cholesterol is integrated into cell membranes, where it helps to maintain membrane structure and fluidity [62,63].

The dysfunction or deficiency of peroxisomes–mitochondria interaction directly affects cholesterol synthesis. A study by Valentina Pallottini and colleagues demonstrated a clear correlation between the activation of HMG-CoA reductase and an increase in reactive oxygen species (ROS) [64]. Peroxisomes are responsible for detoxifying H_2_O_2_ produced during the β-oxidation of fatty acids through the action of catalase. Therefore, if peroxisomal dysfunction occurs, it increases cholesterol production and accumulation via HMG-CoA reductase activation as an indirect effect of ROS accumulation (Figure 3B).

This dysfunctional effect has been demonstrated through deficiencies in PEX genes. In a 2003 study using PEX2 knockout mice, a significant increase in the activity of several cholesterol biosynthetic enzymes (HMG-CoA reductase, IPP isomerase, FPP synthase, and squalene synthase) was observed, along with decreased catalase activity in liver homogenates. This study clearly demonstrated a relationship between ROS accumulation due to reduced catalase activity and increased cholesterol production in the livers of PEX2 knockout mice [65]. This accumulation of ROS can lead to lipid peroxidation, compromising the integrity of organelle membranes, such as those of the mitochondria and ER; direct damage to DNA, contributing to apoptosis or oncogenesis; and damage to mitochondrial respiratory proteins, which impairs the efficiency of the electron transport chain. This results in decreased ATP production and further increases ROS generation, creating a cycle of mitochondrial dysfunction, which leads to the development of different diseases, such as neurodegenerative ones [66].

The dysfunction of mitochondria–peroxisomes and the subsequent increase in ROS levels and cholesterol synthesis also play a crucial role in developing metabolic disorders, such as T2D and MAFLD (Figure 3). Excess cholesterol and lipid accumulation in hepatocytes can lead to the formation of lipid droplets, a hallmark of fatty liver, which can result in lipotoxicity, impaired mitochondrial function, and metabolic disorders, increasing the risk of developing obesity [67]. Additionally, oxidative stress and disrupted lipid homeostasis are closely linked to insulin resistance, a precursor to T2D, which destabilizes the body’s overall glucose metabolism [30].

### 4.2. Estrogen Regulation of Cholesterol Synthesis

Cholesterol synthesis is linked to the production of steroid hormones like E2 (Figure 3A). Estradiol synthesis relies on cholesterol as a precursor, converting it into pregnenolone via the enzyme CYP11A1 in mitochondria, followed by further enzymatic steps that lead to estradiol in the ovaries. In addition, cholesterol can be converted into testosterone and, finally, in estradiol by the action of the aromatase (CYP19) in the ovaries, the most stipulated pathway [68].

E2 regulates cholesterol metabolism by interacting with estrogen-related receptors, which modulate gene expression in lipid biosynthesis. E2 can regulate HMG-CoA reductase expression by stabilizing its mRNA, thereby controlling the rate of cholesterol synthesis in response to the organism’s cholesterol needs, influenced by the action of various hormones, such as FSH, which is regulated by the hypothalamus [69,70].

An experiment with older female rats exposed that age affects their cholesterol metabolism, increasing its synthesis and accumulation in ages in which these rats started an estropausal stage with low estrogen levels, higher levels of plasma cholesterol, and increased activation of HMG-CoA reductase, decreasing the insulin sensitivity at the same time. In addition, a hormonal treatment with E2 in these rats resulted in restoration of normal cholesterol levels, reduced activation of HMG-CoA reductase, and increased LDLR exposure on the plasma membrane [71,72], highlighting the regulatory effect that estrogen has on cholesterol metabolism.

In addition, another study by Schulz and coworkers demonstrated that estradiol-mediated AMPK activation was independent of estrogen receptor ligand engagement, which means that E2 can regulate HMG-CoA reductase activity in the short-term by controlling activation of AMPK [73], supporting the regulatory function proposed before.

## 5. Bile Acids Metabolism

### 5.1. Bile Acids Synthesis and Signaling Function

Bile acids (BAs) are synthesized from cholesterol in the liver and play a crucial role in the digestion and absorption of dietary fats. The metabolism of bile acids begins with the hydroxylation of cholesterol, being their principal substrate, primarily via two pathways: the neutral pathway, in charge of 80–90% of production, and the acidic pathway [74].

After the first steps of both pathways, two principal cholestanoic acids are produced in mitochondria: 3α, 7α-dihydroxycholestanoic acid (DHCA) and 3α, 7α, 12α-trihydroxycholestanoic acid (THCA), which are precursors to bile acids.

Peroxisomes are involved in the β-oxidation of VLCFAs and branched-chain fatty acids, including the side-chain shortening of these bile acid intermediates. These two organelles are indispensable for bile acid metabolism, as they facilitate the conversion of intermediates into their primary forms: taurocholic acid (CA) and glycochenodeoxycholic acid (CDCA) [74,75,76,77,78,79].

Bile acids, traditionally considered essential molecules for lipid digestion and absorption through the forming of micelles that can support the function of lipases in digestion, are now recognized as important signaling agents that regulate various metabolic processes. Specifically, bile acids activate two key receptors: FXR (farnesoid X receptor), a nuclear receptor, and TGR5 (G-protein-coupled bile acid receptor), which is membrane-bound [80].

**Figure 4:**
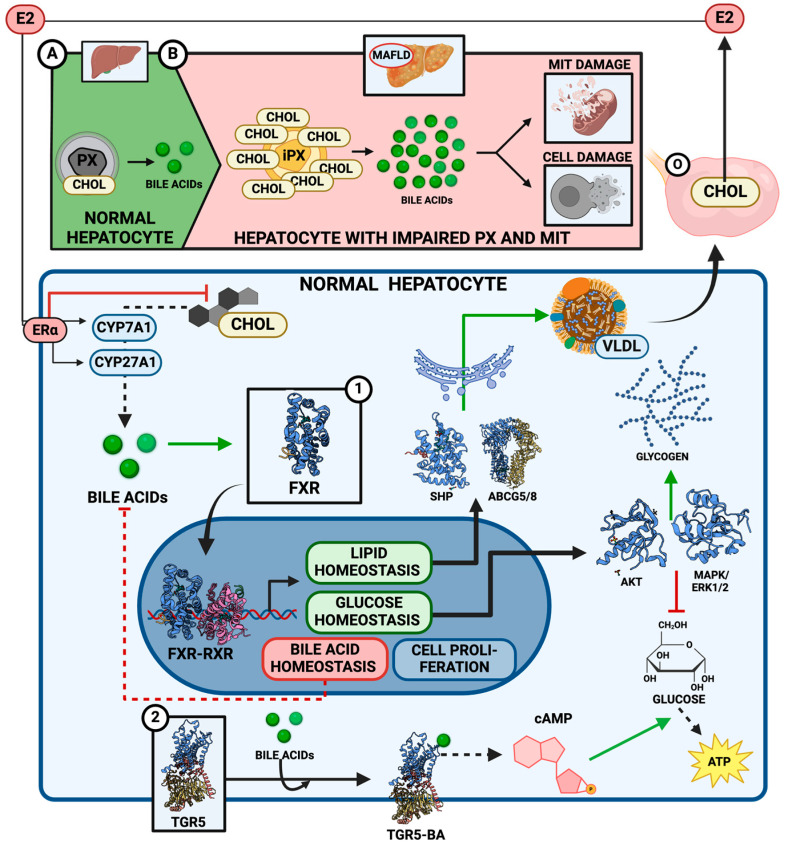
Diagram of bile acid (BA) metabolism and its regulation by 17β-estradiol (E2). (**A**) In a healthy hepatocyte scenario, BA metabolism is primarily regulated by available cholesterol levels, as cholesterol is the main substrate for BA synthesis. BAs act through two key receptors: FXR and TGR5. FXR forms a dimer with RXR in the nucleus, activating the transcription of proteins involved in cell proliferation and regulating lipid, glucose, and bile acid homeostasis. TGR5, on the other hand, is membrane-bound and regulates the cell’s energy balance when activated by bile acids. E2 can control BA production by controlling the cholesterol endogenous synthesis through its estrogen receptor-α (ERα). (**B**) In hepatocytes with impaired peroxisomes (PX) and mitochondria (MIT), excessive cholesterol accumulation leads to uncontrolled bile acid production. This dysregulation causes mitochondrial and cellular damage, ultimately contributing to metabolic dysfunction-associated fatty liver disease (MAFLD) development. Created with www.Biorender.com.

FXR is primarily expressed in the liver, intestines, kidneys, and adipose tissues. Upon activation by bile acids, particularly chenodeoxycholic acid (CDCA), FXR regulates the transcription of genes that maintain lipid and glucose homeostasis. For instance, FXR activation reduces the expression of genes that promote bile acid synthesis (like CYP7A1, the enzyme responsible for the rate-limiting step in bile acid production), thereby exerting feedback inhibition on bile acid synthesis to avoid cytotoxic levels [76,81,82] (Figure 4A).

Additionally, FXR modulates lipid metabolism by regulating genes like SHP (small heterodimer partner) and ABCG5/8 transporters, which are involved in cholesterol excretion. It also plays a crucial role in glucose homeostasis, as FXR activation in the liver suppresses gluconeogenesis and promotes glycogen synthesis, enhancing overall energy balance by the activation of AKT and the mitogen-activated protein kinase/extracellular signal-regulated kinase 1/2 (MAPK/ERK1/2) pathway, which means bile acids may copy the insulin action in glucose metabolism [76,83,84] (Figure 4A).

It was also demonstrated that dysregulation of glucose and insulin levels affects FXR expression and that glucose induction in primary rat hepatocytes promotes the expression of functionally active FXR [85].

TGR5, on the other hand, is expressed in various tissues, including the liver, brown adipose tissue, intestines, and immune cells. When bile acids bind to TGR5, it triggers a signaling cascade that increases intracellular cyclic AMP (cAMP) levels, activating pathways that influence energy expenditure and glucose metabolism. TGR5 activation promotes the release of GLP-1 (glucagon-like peptide-1) from enteroendocrine cells, which improves insulin sensitivity and glucose regulation [86,87] (Figure 4A).

### 5.2. Estrogen Regulation of Bile Acid Metabolism

It is not yet fully understood whether estrogens can regulate bile acid metabolism in the same way they influence cholesterol synthesis. However, it is known that estrogens, particularly estradiol, can interact with and modulate the function of various cytochrome P450 enzymes, such as CYP1B1 [23]. This suggests that estrogens might play a similar role in regulating the activity of CYP7A1 and CYP27A1, key enzymes in bile acid synthesis, though this has not been definitively established (Figure 4A).

Additionally, estrogens are directly linked to cholesterol production, as cholesterol is the precursor for steroid hormone synthesis [68]. This connection is vital in females, where hormonal fluctuations during the menstrual cycle, pregnancy, and menopause significantly impact cholesterol homeostasis [88]. By regulating cholesterol levels, estradiol may indirectly influence the production of bile acids, as cholesterol is also a substrate for bile acid formation [76].

Furthermore, studies have demonstrated a significant correlation between the higher expression of FXR and estrogen receptors (ER), particularly in the context of breast cancer in postmenopausal patients [89], suggesting that the absence of estrogens may reveal the association between FXR and cell proliferation. While this finding is promising, the tumor environment in which this relationship was observed may not fully represent normal physiological conditions, making it difficult to generalize to non-cancerous contexts at this time.

### 5.3. Effect of Dysfunctional Peroxisomes–Mitochondria on Bile Acid Metabolism

Mitochondria and peroxisomes are crucial in converting cholesterol into bile acids, essential for maintaining proper bile acid production and cholesterol homeostasis. When peroxisomal function is impaired, this conversion is disrupted, accumulating unmetabolized bile acid intermediates in the form of cholestanoic acids. This imbalance can result in the development of diseases such as MAFLD, primarily driven by the accumulation of cholesterol and reactive oxygen species (ROS) [90,91,92,93,94] (Figure 4B). Peroxisomes are, at the same time, potential targets for treatments against this disease.

One of the principal metabolic imbalances appears in mitochondria. When bile acid synthesis is disrupted, mitochondria are significantly affected due to the accumulation of toxic metabolites, such as lipids and excess cholesterol [95]. This can lead to an increase in mitochondrial β-oxidation, oxidative stress, and ROS accumulation, which damages the mitochondrial membrane and impairs the function of the electron transport chain, resulting in reduced ATP production [95,96].

This oxidative stress damages mitochondrial proteins, lipids, and DNA, particularly affecting cardiolipin, a crucial phospholipid in mitochondrial bioenergetics and cholesterol mobility, as well [97]. The resulting mitochondrial dysfunction creates a vicious cycle of ROS generation and further damage, contributing to insulin resistance, hepatocyte injury, and obesity [96,98].

Impaired mitochondrial function, together with oxidative stress and disrupted lipid metabolism, significantly increases the risk of apoptosis or necrosis in hepatocytes, accelerating liver damage and the progression of diseases like MASH. The accumulation of excess bile acids exacerbates this process by inducing cellular toxicity, disrupting cellular and mitochondrial membranes, and triggering endoplasmic reticulum stress. This combination of mitochondrial dysfunction and bile acid (BA) toxicity leads to further oxidative stress, DNA damage, and apoptosis. Over time, these disruptions not only drive liver injury but also increase the potential for BA to act as a cancer promoter, adding another layer of complexity to disease progression [99].

Evidence suggests that MASH may induce a shift in classical to alternative metabolic pathways related to bile acids, as indicated by comparing liver samples from affected patients and healthy controls. Specifically, the mRNA of CYP7B1 and hepatic taurine levels were increased, while CYP8B1, cholic (CA), and glycodeoxycholic acid (GDCA) were decreased, which supports the idea of an alteration in BA metabolism due to the appearance of MASH [100].

That is why dysregulated peroxisomes, mitochondria, and, consequently, dysregulated BA metabolism are essential indicators in the pathology of MAFLD, which could develop into more liver injury steps. Several drug candidates targeting BA metabolism intermediates are currently under development [94,101].

## 6. Lipoprotein Metabolism

### 6.1. Key Regulators of Lipoprotein Metabolism in Health and Disease

Lipoproteins are macromolecular complexes that function as the primary lipid transporters in the bloodstream. Their structure consists of a core of hydrophobic lipids, including triglycerides and cholesterol esters, which is surrounded by an amphipathic monolayer of apolipoproteins, phospholipids, and non-esterified cholesterol [102,103].

Based on their physical–chemical features, lipoproteins have been classified into four major classes: chylomicrons, VLDLs, low-density lipoproteins (LDLs), and HDLs. Dietary and hepatically produced triglycerides are conveyed through the bloodstream in the triglyceride-rich lipoproteins (TRLs), chylomicrons, and VLDLs, respectively, while cholesterol is primarily transported in LDLs and HDLs [104,105].

The primary structural protein in TRLs, apolipoprotein B (apoB), is synthesized in apoB-100 and apoB-48. While apoB-48 is produced in the intestine and aids in the development of chylomicrons, apoB-100 is expressed in the liver and is necessary for the formation of VLDL [106].

Hepatically produced triglycerides are transported by VLDLs to peripheral tissues for use. The process of VLDL assembly is intricate and strictly controlled. This pathway initiates with the co-translational lipidation of the developing apoB polypeptide in the endoplasmic reticulum (ER), a process facilitated by microsomal triglyceride transfer protein (MTTP) [107].

Several studies support the view that the rates at which VLDL1 and VLDL2 are produced in the liver are separately regulated. Elevated hepatic triglyceride levels induce the production of VLDL1, perhaps as a protection mechanism against the harmful effects of triglyceride overload on hepatocytes. Insulin suppresses VLDL1 release from the liver both directly (postprandially in response to the presence of circulating intestinally derived TRLs) and indirectly (by lowering non-esterified fatty acid (NEFA) flow into the liver). Both direct and indirect regulatory pathways are compromised in individuals with insulin resistance, fatty liver, and T2D [108,109,110]. On the other hand, the synthesis of VLDL2 is more directly related to cholesterol metabolism. When endogenous cholesterol synthesis is induced, the rate of VLDL2 secretion rises, presumably to control the amount of cholesterol in the liver [111,112].

There is now proof that the intestine’s production of TRLs does not only function as a response to the presence of food in the gut. Indeed, several hormones modulate chylomicron and apoB-48-VLDL release. Like their effects on the liver’s VLDL secretion, insulin and NEFAs both control the assembly and release of chylomicrons. In insulin-sensitive individuals, insulin inhibits the intestinal secretion of apoB-48-containing lipoproteins, whereas insulin resistance appears to play a significant role in the elevated synthesis of chylomicron and apoB-48-VLDLs observed in T2D [113,114].

In HepG2 cells, insulin has been shown to regulate MTTP by decreasing the transcription of its gene in a dose- and time-dependent fashion [115]. Insulin specifically regulates MTTP through the MAPK/ERK cascade, inhibiting the MTTP gene’s transcription. Remarkably, MAPK/ERK and insulin both suppress MTTP gene transcription while upregulating the transcription of the LDL receptor (LDL-R) gene [116]. Several studies appear to suggest that the activation of MAPK/ERK in the liver possesses beneficial effects on the plasma lipid profile since it can both trigger the expression of LDL-R and block the expression of the hepatic MTTP gene, thus promoting apoB clearance and reducing apoB secretion, respectively. Nevertheless, the advantageous role of hepatic MAPK/ERK appears to be disrupted in animals suffering from T2D [117].

Lipoprotein lipase (LPL) also plays an essential part in lipid metabolism. This enzyme, which is mainly expressed in the heart, skeletal muscle, and white adipose tissue (WAT) but not in the liver, catalyzes the hydrolysis of circulating triglycerides that compose TRLs. Since LPL is the rate-limiting factor for both tissue uptake of fatty acids and plasma triglyceride clearance, its activity is meticulously regulated through a variety of strategies, which involve insulin, apolipoproteins, and angiopoietin-like (ANGPTL) proteins [118,119].

Insulin promotes LPL activity in adipose tissue after a meal. However, in insulin-resistant individuals, this regulatory mechanism is severely compromised in the postprandial state. Indeed, individuals with insulin resistance and T2D patients exhibit decreased LPL activity in adipose tissue and skeletal muscle. Furthermore, diabetic dyslipidemia has been linked to low LPL function and elevated plasma triglycerides [120,121].

**Figure 5:**
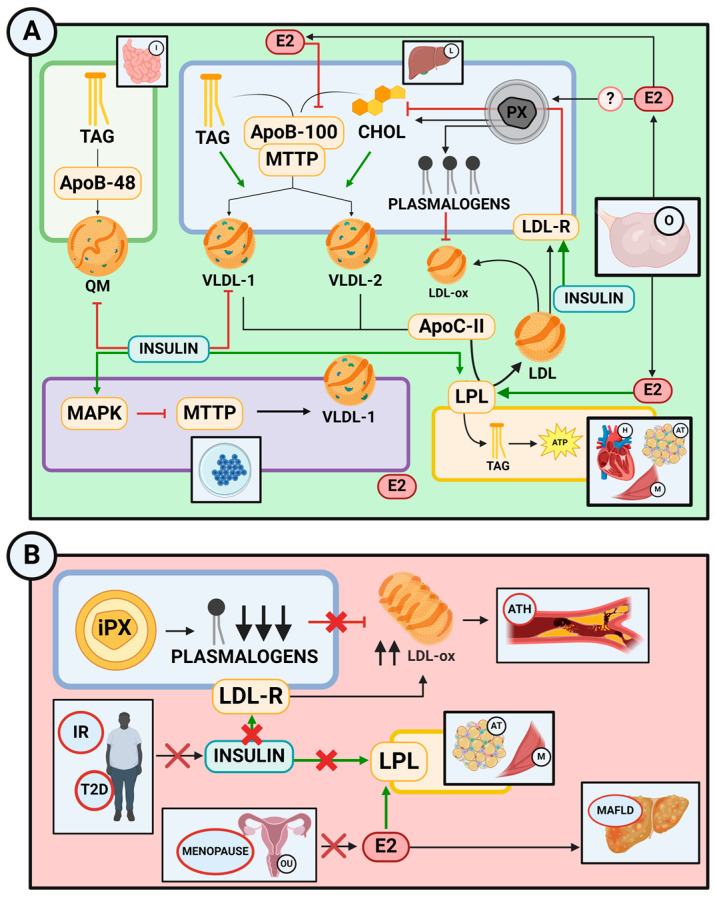
Lipoprotein metabolism and its implication in health and disease. (**A**) In healthy individuals, insulin, estrogens, and peroxisomal plasmalogens, among others, work together to tightly regulate lipoprotein synthesis and secretion, their metabolism in the bloodstream, and their clearance. (**B**) Disruption of lipoprotein metabolic pathways occurs in various conditions, including insulin resistance (IR), type 2 diabetes (T2D), menopause, and atherosclerosis. I: intestine; L: liver; E2: 17β-estradiol; TAG: triacylglyceride; QM: chylomicron; CHOL: cholesterol; LDL-R: low-density lipoprotein receptor; VLDL: very-low-density lipoprotein; PX: peroxisome; O: ovary; H: heart; AT: adipose tissue; M: muscle; iPX: impaired peroxisome; ATH: atherosclerosis; LPL: lipoprotein lipase; OU: ovaries and uterus; MAFLD: metabolic dysfunction-associated fatty liver disease. Created with www.Biorender.com.

Peroxisomes mainly impact systemic metabolism by acting as lipid metabolic organelles, producing ether lipids, such as plasmalogens, oxidizing VLCFA, and regulating H_2_O_2_ balance. Indeed, emerging evidence points out that these organelles play a significant role in maintaining energy homeostasis in collaboration with mitochondria and that impairment of peroxisomal functions increases the risk of several metabolic conditions, including obesity, T2D, and hepatic steatosis [7,10,122,123] (Figure 5B).

By secreting plasmalogens, peroxisomes appear to have a preventive effect against atherosclerosis, whereas impairments in peroxisomal functioning in the liver appear to be associated with alterations in lipid metabolism [124].

A diagram of lipoprotein metabolism can be seen in Figure 5A.

### 6.2. Estrogen’s Protective Effects on Lipoprotein Failure Metabolism Diseases

Many aspects of sex differences in the risk of atherosclerotic heart disease can be associated with the divided actions of estrogens in muscle, adipose tissue, and the liver [11]. In fact, it is believed that the effects of estrogens on lipid metabolism account for 25 to 50% of their benefits when administered to postmenopausal women. For instance, the main circulating estrogen in humans, E2, decreases the rate at which apoB-100 is produced in the liver and speeds up the synthesis of LPL in adipose tissue [125].

Men are more likely than women to have higher levels of obesity-related increased liver production of VLDL particles. Both the synthesis of more triglyceride-rich VLDLs in women and faster VLDL clearance rates contribute to reducing plasma VLDL levels associated with obesity in women [126,127]. In this scenario, estrogens are essential regulators of lipid metabolism, and it has been demonstrated that estrogen-signaling defects promote hepatic insulin resistance and liver triglyceride buildup. Ovariectomy results in an accumulation of hepatic triglycerides in mice, which is comparable to what occurs in postmenopausal women [128,129]. Additionally, a chemical model of menopause has also been shown to induce dyslipidemia, fatty liver, and insulin resistance in mice [19].

In a mouse model with ERα-deficiency in hepatocytes, the capacity of estrogens to decrease liver steatosis has been demonstrated to be lost, suggesting that estrogens act directly in the liver to lower triglycerides through ERα [130]. Furthermore, the fact that estrogen signals adipose tissue to restrict the production of serum fatty acid (FA) in response to insulin and skeletal muscle to enhance FA oxidation, reducing FA delivery to the liver, suggests that this is probably responsible for some of the indirect protective effects of estrogen on the liver. Thus, poor coordination of the liver, muscle, and adipose tissues concerning the flow of FA and estrogen regulatory mechanisms contributes to dyslipidemia and aberrant glucose levels associated with obesity [131,132,133].

Related to cholesterol synthesis and metabolism, animal studies have revealed that the protein levels of HMG-CoA reductase in female or E2-treated male rats are lower than in mature untreated males. Additionally, pharmacological doses of estrogens in rats raise the levels of hepatic LDL-R mRNA and protein, which results in an important increase in the clearance of plasma LDL and a significant reduction in plasma cholesterol [134,135].

Interestingly, the sex difference in cardiovascular risk may be influenced by regulating HDL hepatic absorption by estrogens. Indeed, compared to male mice fed a Western diet, female mice showed higher total-body reverse cholesterol transport (RCT), and the total-body RCT was impaired in female mice after ERα removal. Moreover, according to several studies, estrogens increase the expression of the HDL receptor and scavenger receptor class B type 1 (SR-B1) and facilitate the uptake of HDL-cholesterol in the peripheral tissues [136,137].

Estrogens are also involved in preserving healthy mitochondrial function [138]. Indeed, E2 protects the liver from oxidative damage, a crucial feature of the pathogenesis of T2D. It was shown that in comparison to healthy female mice, males and ovariectomized females presented a higher profile of oxidative stress, including higher production of H2O2 and lower levels of glutathione [139]. Collectively, these findings suggest that estrogen’s antioxidant activity prevents the onset of T2D in females by protecting against oxidative stress and the resulting mitochondrial damage [140]. Additionally, the underlying causes of cardiac dysfunction linked to metabolic syndrome being more prevalent in menopausal women involve increased oxidative stress, which harms mitochondria, activates mitochondrial apoptotic signaling pathways, and impairs cardiomyocyte contractile performance [141] (Figure 5).

Future research ought to investigate a link between the possible estrogen regulatory actions on peroxisomes and, as has been demonstrated for mitochondria, the protective antioxidant effects of these hormones for assuring peroxisomal function maintenance.

## 7. Conclusions

This review highlights the crucial role of estrogens, especially E2, in regulating peroxisomal and mitochondrial metabolism, which is essential for lipid regulation and cholesterol homeostasis, particularly in female obesity and type 2 diabetes (T2D). Disruptions in these pathways, such as those caused by estrogen deficiency in postmenopausal women, lead to lipid accumulation and increased risk of metabolic disorders. Estrogens influence liver function, modulate lipid metabolism through nuclear receptors like PPARs, and offer protective effects against insulin resistance and fat deposition. This review also emphasizes the therapeutic potential of hormone replacement therapy and PPAR agonists in restoring metabolic balance. Understanding these molecular mechanisms is crucial in developing treatments for metabolic diseases like obesity, MAFLD, and T2D.
